# HSC70 is required for infectious bursal disease virus (IBDV) infection in DF-1 cells

**DOI:** 10.1186/s12985-020-01333-x

**Published:** 2020-05-06

**Authors:** Chunbo Chen, Ying Qin, Kun Qian, Hongxia Shao, Jianqiang Ye, Aijian Qin

**Affiliations:** 1 Ministry of Education Key Laboratory for Avian Preventive Medicine, Yangzhou University, Yangzhou, Jiangsu 225009 China; 2 Jiangsu Co-innovation Center for Prevention and Control of Important Animal Infectious Diseases and Zoonoses, Yangzhou, Jiangsu 225009 China; 3 Joint International Research Laboratory of Agriculture and Agri-Product Safety, the Ministry of Education of China, Yangzhou University, Yangzhou, Jiangsu 225009 China

**Keywords:** Infectious bursal disease virus, HSC70, DF-1 cells, Virus infection

## Abstract

**Background:**

Infectious bursal disease (IBD) is a highly contagious infectious disease that causes severe immunosuppression and damage to the bursa of Fabricius in chickens. Several proteins involved in IBD virus (IBDV) infection, such as surface immunoglobulin M, integrin, annexin A2 and chicken heat shock protein 90, have been identified. However, the main protein that plays key roles in virus infection has not yet been confirmed.

**Methods:**

DF-1 cell line was transfected with the pcDNA-VP2 plasmid and analyzed by immunofluorescence assay. The proteins reacted with VP2 of IBDV in DF-1 cells were pulldown with the monoclonal antibody and identified by mass spectrometry. Heat shock cognate protein 70 (HSC70), one of these proteins, was selected to be investigated in the function in IBDV infection by specific antibody and its inhibitor.

**Results:**

The DF-1 cell line was transfected with the pcDNA-VP2 plasmid, and expression of IBDV VP2 in DF-1 cells was confirmed by immunofluorescence assays. Heat shock cognate protein 70 (HSC70) was one of the proteins identified by coimmunoprecipitation using a monoclonal antibody (2H11) against VP2 and mass spectrometry analysis. IBDV infection in DF-1 cells was strongly inhibited by both an anti-HSC70 antibody and a HSC70 inhibitor (VER155008).

**Conclusion:**

These results suggest that HSC70 may be an essential factor for IBDV infection.

## Background

Infectious bursal disease virus (IBDV), a birnavirus belonging to the Birnaviridae family, is a non-enveloped virus that consists of 5 proteins, namely, VP1, VP2, VP3, VP4, and VP5 [1]. VP2 is the main host protective antigen and a capsid protein [2, 3]. As a severe immunosuppressive disease prevalent in chickens, IBDV has resulted in high economic loss in the poultry industry [4]. Many researchers to date have studied the pathogenesis of the disease caused by IBDV. For example, Hirai et al. found that surface immunoglobulin M is a receptor on LSCC-BK3 cells [5], and Lin et al. showed that chicken HSP90 is a component of the putative cellular receptor complex essential for IBDV entry into DF-1 cells [6]. The alpha 4 beta 1 integrin is another putative receptor, was verified by Delgui et al. on BALB/c 3 T3 cells [7]. In addition, using a virus overlay protein-binding assay (VOPBA) with DF-1 cells, Ren et al. demonstrated that chicken annexin A2 is positively related to viral infection [8]. All of these proteins have been found to promote viral infection.

Two proteins, HSP70 and HSC70, from the HSP70 family are highly conserved and composed of an N-terminal ATPase domain, a substrate-binding domain, and a C-terminal domain [9–11]. HSP70 and HSC70 are not only present in the cytoplasm but are also found on the cell surface of various cells, such as arterial smooth muscle cells, epidermal cells and tumor cells [12–14]. The HSP70 family is an important group of the molecular chaperone family that has been reported to play a crucial role in different stages of the viral life cycle, including in virus entry, virion disassembly/assembly, viral genome replication, and virus particle formation [15]. Rotavirus is a typical nonenveloped virus that exploits surface-exposed HSC70 to enter epithelial cells at a post attachment step [16]. Similarly, coxsackievirus A9 interacts with GRP78, a member of the HSP70 family. GRP78 promotes viral attachment on target cells [17]. In BHK-21 and HeLa cells, HSP70 is associated with the adenovirus capsid protein that interacts with HSP70 after penetration [18]. Furthermore, HSP70 and HSP40 interact with the herpes simplex virus type-1 initiator protein (UL9); hence, these two heat shock proteins are involved in the initiation of viral DNA replication [19]. Regarding human papillomavirus, DNA synthesis and genome replication are enhanced by HSP70 and HSP40 [20]. Some research has shown that HSC70 isoform D is strongly involved in clathrin-mediated endocytosis, especially at the late stage of Japanese encephalitis virus entry into C6/36 cells [21]. In this paper, we identified HSC70 as VP2 interacting protein using immunoprecipitation and mass spectrometry, and its function in IBDV infection was investigated.

## Materials and methods

### Virus, cells, and antibodies

The DF-1 cell line, an immortalized cell line of chicken embryo fibroblasts that is susceptible to many isolated avian viruses, was provided by Dr. Lucy Lee, USDA. The cells were cultured in Dulbecco’s modified Eagle’s medium (DMEM) containing 5% fetal bovine serum (FBS) at 37 °C in a 5% CO_2_ atmosphere. A specific monoclonal antibody 2H11 against IBDV was prepared in our previous work [22]. The IBDV Q strain is an IBDV isolated from the infected chickens. This strain proliferated in DF-1 cells with a multiplicity of infection (MOI) of 1 and was cultured at 37 °C in DMEM (Gibco, China) with 1% FBS. After the cytopathic effect (CPE) completely appeared, approximately 36 h, the cells and supernatants were collected. Viruses were obtained by 3 cycles of freezing and thawing, and titers were determined using the Reed-Muench method.

### Cell protein extraction

DF-1 cells transfected with the plasmid pcDNA-VP2 or with the pcDNA vector were separately cultured in DMEM containing 5% FBS at 37 °C in 5% CO_2_. After 72 h, the cells were washed 3 times with precooled phosphate-buffered saline (PBS) and harvested using a disposable cell scraper. The cells were centrifuged (13,200 rpm, 5 min), and lysis buffer (containing a cocktail of protease inhibitors, 150 mM NaCl, 1% NP-40, 25 mM Tris, and 5% glycerol) was added. The cell pellets were dispersed by repeat pipetting, placed on ice for 30 min, and centrifuged at 13,200 rpm/min for 25 min. Protein A + G agarose or control resin was added to the supernatants and the mixture was softly shaken at 37 °C for 3.5 h. The samples were centrifuged at 1000×*g* for 5 min, the supernatants were collected.

### Coimmunoprecipitation

Coimmunoprecipitation assays were performed using a coimmunoprecipitation crosslinking kit (Thermo Fisher Scientific, Pierce Biotechnology, IL, USA) according to the manufacturer’s instructions. The kit enables the isolation of native protein complexes from a lysate or other complex mixture by directly immobilizing purified antibodies onto an agarose support. In this study, supernatants containing cell protein extracts were incubated with the monoclonal antibody 2H11, which is specific for the IBDV VP2 protein. Native proteins isolated using the kit were resuspended in 5 × SDS sample buffer, boiled for 10 min, and subjected to 10% SDS-PAGE. After electrophoresis, the gels were stained with a silver staining kit (Thermo Fisher Scientific, Pierce Biotechnology, IL, USA). The differentially abundant protein bands compared to those in the negative control were excised and identified by mass spectrometry.

### Mass spectrometric analysis

As indicated above, differentially abundant proteins were identified by comparison of the protein bands of the experimental and the control groups. The differential proteins were excised and sent to Shanghai Zhongke New Life Biotechnology Co., Ltd. for mass spectrometry analysis.

The gel samples were added to approximately 200-400 μL of ACN/100 mM NH_4_HCO_3_, washed and decolored to transparency, and freeze dried after removal of the supernatants. The samples were combined with DTT and incubated at 56 °C for 30 min, after which the DTT solution was replaced with 200 mM IAA prior to incubation in the dark for 20 min. The supernatants were removed, and 100 mM NH_4_HCO_3_ was added to the samples followed by incubation at room temperature for 15 min. The NH_4_HCO_3_ solution was replaced with 100% ACN, and the samples were incubated for 5 min, absorbed and freeze dried. Trypsin solution (2.5–10 ng/μL) was added to the mixture and incubated at 37 °C for approximately 20 h. The original solution was transferred to a new Eppendorf tube, and 100 μL of extraction solution (60% ACN/0.1% TFA) was added to the gel. After ultrasonication for 15 min, the samples were combined with the enzymatic hydrolysate and lyophilized. A solution of 0.1% formic acid was added to the samples for resolving, and the samples were collected by filtration through a 0.22-μm membrane.

The mass-charge ratios of the polypeptide fragments were determined using a full scan method each time. Bioworks Browser 3.3 software was employed to retrieve the corresponding database for the mass spectrometry test raw file to obtain the protein identification results. The retrieval parameters were as follows: database: uniprot; taxonomy: *Gallus gallus*; enzyme: trypsin; dynamical modifications: oxidation (M); fixed modifications: carbamidomethyl (C); max missed cleavages:2; peptide charge state: 1 + , 2 + , and 3+; proteomics tools: 3.1.6. Filter by Delta CN:charge =1 Delta CN ≥ 0.1; charge =2 Delta CN ≥0.1; charge =3Delta CN ≥0.1; Filter by Xcorr:charge =1 Xcorr ≥1.9; charge =2 Xcorr≥2.2; charge =3 Xcorr≥3.75.

### Indirect immunofluorescence assay (IFA) and confocal microscopy

DF-1 cells were cultured on glass cover slips, fixed on glass with 3% paraformaldehyde for 20 min at room temperature, and washed 3 times with PBS. The cells were then incubated with a membrane disrupting solution containing 0.25% Triton X-100 at room temperature for 5 min. These samples were blocked with 2% bovine serum albumin (BSA) at 37 °C and incubated for 45 min. An anti-HSC70 antibody or normal immunoglobulin G (IgG) was diluted to 1:100 as the primary antibody, and FITC-conjugated goat anti-mouse IgG was used as the secondary antibody. The samples were incubated with the two antibodies for 45 min and then observed using a laser scanning confocal microscope.

### Western blotting

Protein extracts of DF-1 cells transfected with pcDNA-VP2 or the pcDNA vector were separated by 10% SDS-PAGE. The proteins were then transferred to a nitrocellulose membrane, which was then blocked with 5% nonfat milk in Tris-buffered saline (TBS) buffer at 4 °C overnight and washed 2 times with TBS. The proteins were incubated with a primary antibody at 37 °C for 2 h and subsequently with a secondary antibody at 37 °C for 2 h. After washing 3 times, detection using chemiluminescence reagents was performed.

### HSC70 inhibitor (VER-155008) inhibition assay

A VER-155008 stock liquor was prepared by dissolving VER-155008 (Sigma) in dimethyl sulfoxide (DMSO) and diluted with water into a series of concentrations. DF-1 cells were subcultured in 24-well plates for 18–24 h. The inhibitor (VER-155008) was added to the cell culture medium at a final concentration of 5 μmol/L, and the medium was added to the cells in the incubator at 37 °C for 6 h. The inhibitor was removed from the culture medium by washing twice with sterile PBS. DF-1 cells were cultured in fresh medium containing 1% FBS and infected with IBDV at an MOI of 0.1 for 2 h. After washing 3 times with PBS, medium containing 1% FBS was added to cells. Cell morphology tests, 50% tissue culture infectious dose (TCID_50_) determination, western blotting and IFA were performed at 24, 48, and 72 h, and a positive control was performed synchronously at the indicated time points. The inhibitory effects of different concentrations of the inhibitor were also assessed. DF-1 cells were cultured in 6-well plates for 18 h and incubated at 37 °C for 6 h with the cell culture medium containing different diluted concentrations of the inhibitor (5 μM, 0.5 μM, 0.05 μM and 0.005 μM), which was then replaced with fresh culture medium. DF-1 cells were inoculated with IBDV at an MOI of 0.1 and cultured for 72 h. The CPE, viral titer and protein expression level were measured.

### TCID_50_ assay

The effect of inhibition was determined by detecting the viral titer (TCID_50_) of IBDV. The detailed steps are as follows. DF-1 cells were trypsinized and plated in 96-well plates (3 × 10^4^ cells per well) and placed in a CO_2_ incubator at 37 °C for 18 h. The supernatants from antibody-blocking assays or HSC70 inhibitor assays were diluted 10^1^- to 10^10^-fold with cell culture medium containing 1% FBS. The culture medium in 96-well plates was discarded and replaced by the fresh medium diluted described above. Every diluted concentration of the inhibitor was added to 8 duplicate wells for DF-1 cells infection. After incubation for 2 h at 37 °C, the medium of each well was replaced by fresh medium containing 1% FBS. After 1 week, the cells were observed using an inverted microscope, and a well with CPE was selected. The TCID_50_ was determined by the CPE and calculated by the method of Reed and Muench.

### Antibody inhibition assay

DF-1 cells were cultured in 48-well plates, and each well was seeded with 1.3 × 10^5^ cells. After incubation for 24 h, anti-HSC70 antibodies at different concentrations (6 μg/mL, 12.5 μg/mL, and 25 μg/mL) were added. A normal control IgG was used at a final concentration of 25 μg/mL. A positive control group without any antibody pretreatment was normally infected with IBDV. The DF-1 cells were incubated with anti-HSC70 antibodies at different concentrations at 37 °C for 2 h and subsequently challenged with IBDV at an MOI of 0.01 in fresh medium. At the same time, DF-1 cells were incubated with the normal control IgG at a concentration of 25 μg/mL at 37 °C for 2 h, and then infected with IBDV at an MOI of 0.01 in fresh medium. After incubation for 2 h, unbound viruses were removed by extensive washing with PBS. The infected cells were cultured in medium containing 1% FBS. All infected cells pretreated with the anti-HSC70 antibody were examined by western blotting, IFA, and viral titer (TCID_50_) determination after 48 h. Infected cells pretreated with normal control IgG and the positive control group were also analyzed.

## Results

### Many proteins react with IBDV VP2

**Fig. 1:**
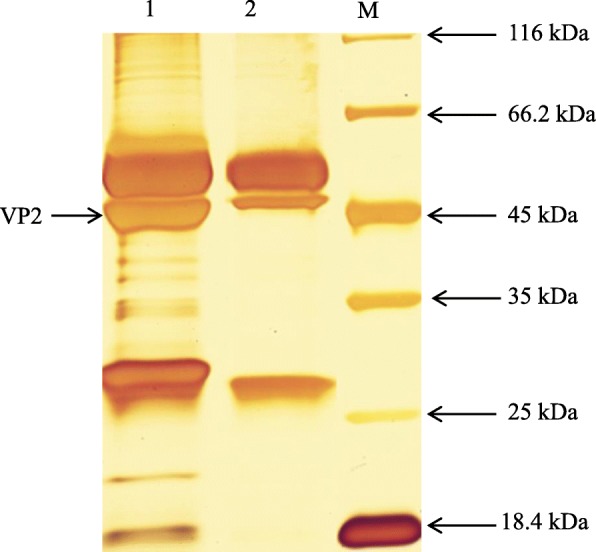
pcDNA-VP2-DF-1 cell proteins were subjected to coimmunoprecipitation and silver staining. Lane 1, Some protein bands were coimmunoprecipitated with monoclonal antibody 2H11; lane 2, Few protein bands were coimmunoprecipitated with normal control IgG; lane 3, protein marker

The proteins of DF-1 cells transfected with the plasmid pcDNA-VP2 or DF-1 cells transfected with the pcDNA vector (control) were extracted and reacted with a monoclonal antibody against IBDV VP2. The proteins from the coimmunoprecipitation elution were separated by 10% SDS-PAGE and silver stain. Many protein bands were found in the experimental group, but only a few bands were observed in the control group (Fig. 1). This discrepancy means that many proteins were able to interact with IBDV VP2 in DF-1 cells.

### HSC70 interacts with VP2 according to mass spectrometry analysis

**Fig. 2:**
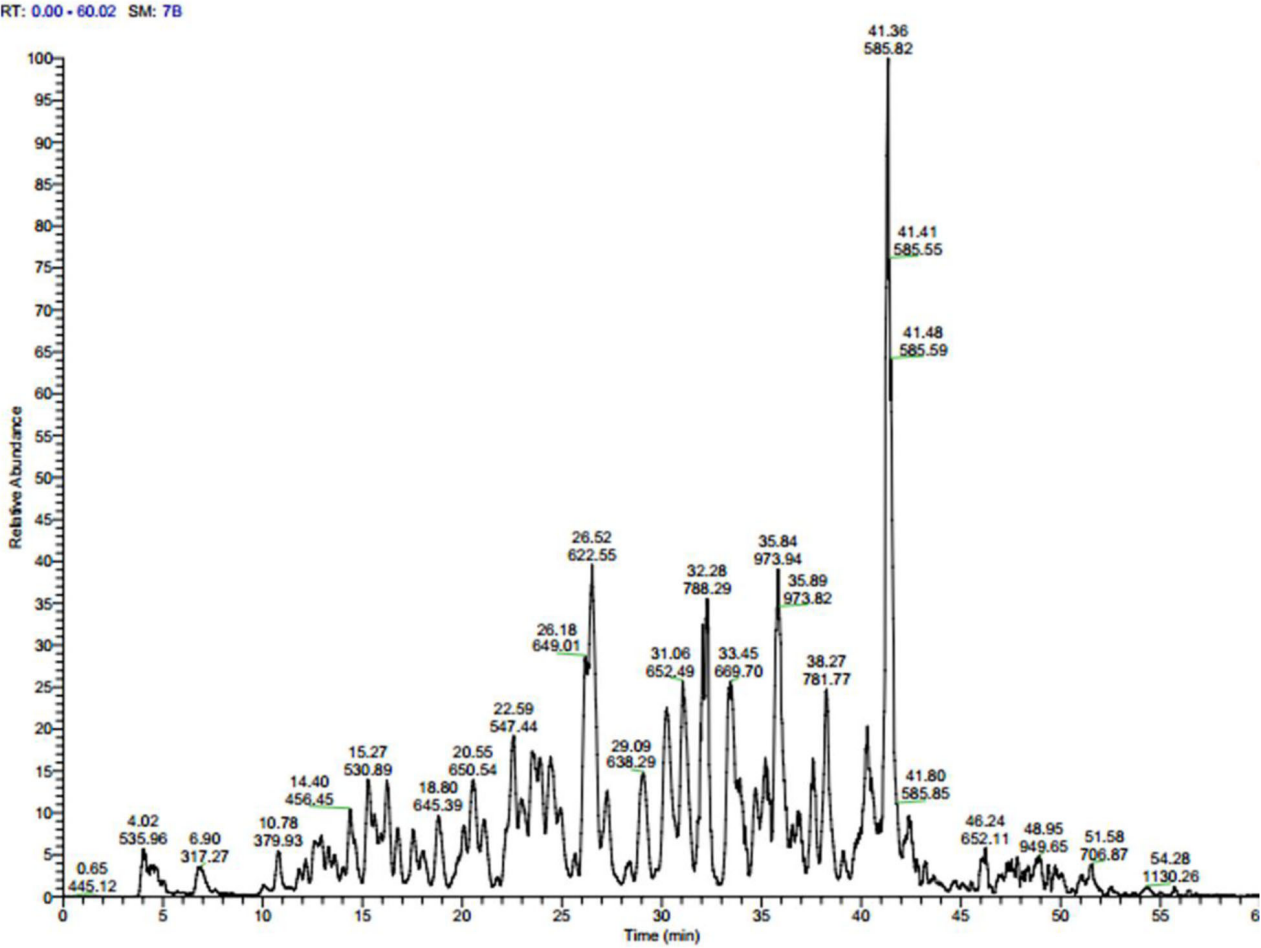
MALDI-TOF analysis result of proteins bound by IBDV VP2 using coimmunoprecipitation. The mass-charge ratios of the polypeptide fragments were determined using a full scan method. The main mass peaks were observed

Mass spectrometry results (Fig. 2) revealed nearly 45 types of protein molecules able to bind to the VP2 protein. As shown in Table 1, the identified proteins belong to 3 main categories: cytoskeletal-associated proteins, enzyme-related proteins, and cell regulatory-related proteins. HSC70 (approximately 70 kDa) was identified among these differential proteins. This finding indicated that VP2 protein expressed in DF-1 cells might specifically bind to HSC70.

**Table 1:** Proteins identified by mass spectrometry

Protein group	Protein number	Protein name	Accession no.	Coverage percent	Unique peptide count
Cytoskeletal- associated proteins	1	Myosin-9	sp**|**P14105**|**	31.55%	122
	2	Vimentin	tr**|**F1NJ08**|**	68.48%	154
	3	Nonmuscle myosin heavy chain	tr**|**Q789A6**|**	16.80%	49
	4	Actin, cytoplasmic type 5	sp**|**P53478**|**	46.54%	30
	5	Actin, aortic smooth muscle	sp**|**P08023**|**	24.40%	12
	6	Alpha tropomyosin of brain	tr**|**Q91005**|**	20.76%	20
	7	Paranemin	tr**|**O57613**|**	6.18%	14
	8	Beta-tropomyosin	tr**|**Q05705**|**	21.77%	12
	9	Unconventional myosin-Ic	sp**|**Q5ZLA6**|**	4.96%	9
	10	Ovalbumin	sp**|**P01012**|**	13.73%	10
	11	Myosin-11	sp**|**P10587**|**	2.09%	7
	12	heat stable cytoskeletal protein	tr**|**Q9PSS9**|**	42.31%	3
	13	Filamin	tr**|**Q90WF1**|**	1.72%	5
	14	Unconventional myosin-VI	sp**|**Q9I8D1**|**	3.06%	3
	15	F-actin-capping protein	sp**|**P14315**|**	14.08%	2
	16	Tubulin alpha-1	sp**|**P02552**|**	8.01%	2
	17	EF hand-containing protein 1	tr**|**Q49B65**|**	7.14%	5
	18	protein epsilon	sp**|**Q5ZMT0**|**	7.45%	2
	19	FERM, RhoGEF and pleckstrin domain-containing protein 1	sp**|**F1P065**|**	1.72%	1
	20	Spectrin alpha chain, nonerythrocytic 1	sp**|**P07751**|**	0.69%	1
	21	Histone	sp**|**P84229**|**	23.53%	1
	22	Coronin	tr**|**Q5ZI60**|**	6.12%	1
Enzyme-related proteins	1	Serine/threonine-protein phosphatase	sp**|**P62207**|**	10.70%	6
	2	DNA ligase	tr**|**F1P394**|**	2.22%	2
	3	Protein phosphatase 1 regulatory	sp**|**Q90623**|**	1.00%	2
	4	ATP synthase subunit alpha	tr**|**F1NI22**|**	3.98%	2
	5	GMP reductase	tr**|**F1NWY8**|**	4.70%	1
	6	Isoleucine-tRNA ligase, mitochondrial	sp**|**Q5ZKA2**|**	1.70%	1
	7	Phosphatidylinositol 4-phosphate 5-kinase type-1	sp**|**Q5ZJ58**|**	1.85%	1
	8	Protein phosphatase 1 regulatory subunit 21	sp**|**Q5ZL12**|**	1.16%	1
	9	Nonspecific serine/threonine protein kinase	tr**|**F1NAH4**|**	0.70%	1
	10	Succinate dehydrogenase, mitochondrial	sp**|**Q9YHT2**|**	4.83%	1
	11	Phosphoinositide phospholipase C	tr**|**E1C7E3**|**	0.65%	1
	12	Protein-tyrosine-phosphatase	tr**|**F1NA27**|**	1.84%	1
	13	Alpha-enolase	sp**|**P51913**|**	5.07%	1
Cell regulatory-related proteins	1	Heat shock cognate 71 kDa protein	sp**|**O73885**|**	7.89%	6
	2	Elongation factor 1	sp**|**Q90835**|**	4.98%	4
	3	Stress-70 protein, mitochondrial	sp**|**Q5ZM98**|**	5.19%	3
	4	Protocadherin-15	sp**|**Q0ZM14**|**	0.74%	2
	5	Tumor suppressor BRCA2	tr**|**Q8UW79**|**	0.53%	2
	6	60S ribosomal protein L6	tr**|**Q8UWG7**|**	5.03%	2
	7	Neuronal acetylcholine receptor	sp**|**P26152**|**	3.08%	1
	8	Elongator complex protein 3	sp**|**Q5ZHS1**|**	3.30%	1
	9	Monocarboxylate transporter 4	sp**|**P57788**|**	2.11%	1
	10	Lamin-B1	tr**|**F1NAM2**|**	2.00%	1

### An anti-HSC70 antibody blocks IBDV infection

**Fig. 3:**
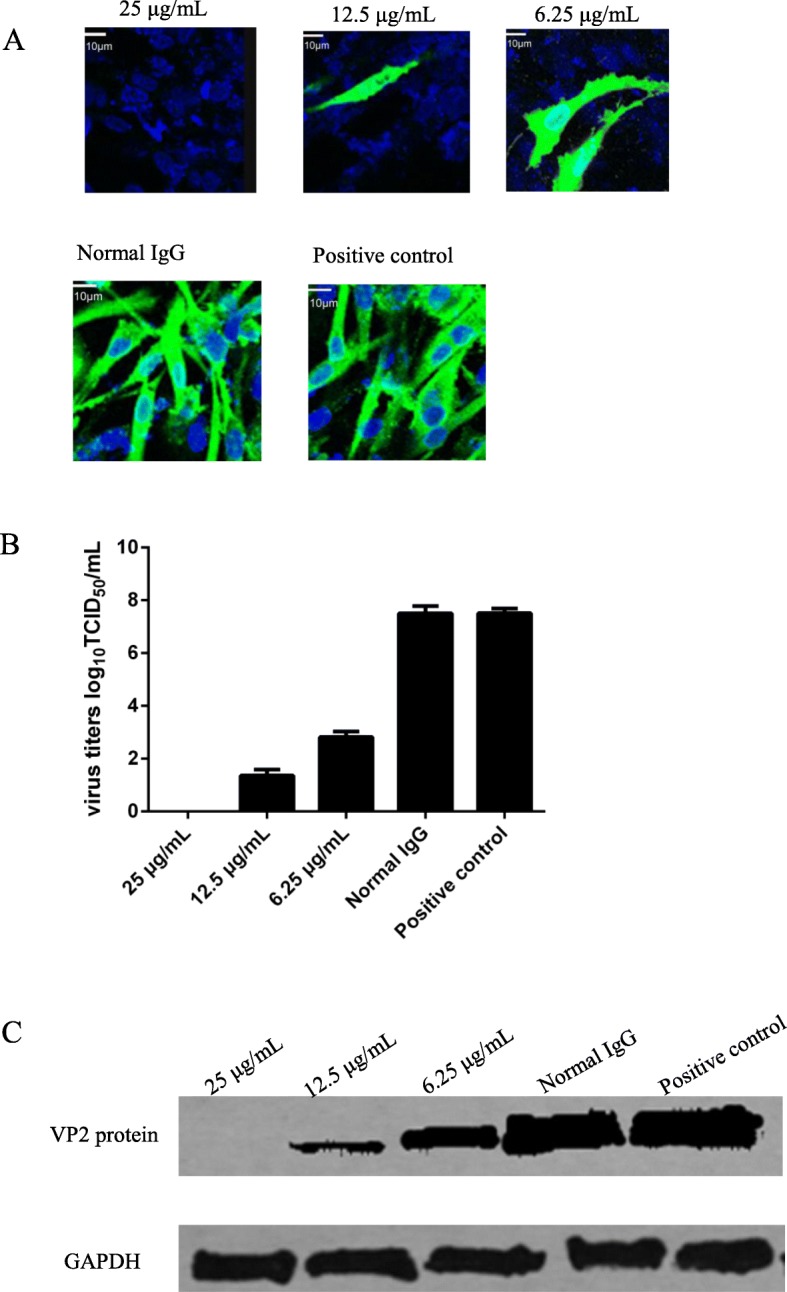
IBDV infection in DF-1 cells blocked by anti-HSC70 antibody at different concentrations. **a**: IFA results showed DF-1 cells pretreated with the antibody to HSC70. The higher concentration of the antibody, the blocking effect better. Normal IgG: same isotype of IgG for control. Positive control: DF-1 cells infected with IBDV. **b**: The viral titers in DF-1 cells after pretreatment with the anti-HSC70 antibody at different concentrations. **c**: Western blot results of the protein expression level of VP2 in DF-1 cells treated with/without antibody to HSC70. Lane 1–3: the protein expression levels treated with anti-HSC70 antibody at concentration of 25 μg/mL, 12.5 μg/mL and 6.25 μg/mL respectively; lane 4: normal IgG as control; lane 5: DF-1 cells infected with IBDV as positive control; GAPDH: the internal reference control. The relative expression level of VP2 was quantified by normalizing GAPDH control. After data analysis, the values of VP2/GAPDH are 0, 0.537, 1.425, 3.329, and 3.345 from left to right

To investigate the function of HSC70 in IBDV infection, we used an antibody against HSC70 to block IBDV infection in DF-1 cells. IFA results (Fig. 3a) showed that IBDV infection was blocked by the antibody against HSC70. IBDV did not grow in DF-1 cells pretreated with the antibody against HSC70, with no specific bright green fluorescence in IFA. In contrast, IBDV could grew very well in the no-antibody-treated or normal IgG (same isotype)-treated groups, which exhibited specific bright green fluorescence. The blocking effects of the antibody against HSC70 were dose dependent when we used different concentrations of the antibody. Although no green fluorescence was observed when we added the antibody at 25 μg/mL, green fluorescence was observed at 6.25 μg/mL.

Viral titers were also determined when the DF-1 cells were blocked with the anti-HSC70 antibody. The results showed that (Fig. 3b) virus replication was decreased when DF-1 cells were pretreated with the anti-HSC70 antibody. A negative correlation between viral titer and antibody concentration was observed in the supernatants of DF-1 cells pretreated with different concentrations of the anti-HSC70 antibody. The same results were found via western blotting (Fig. 3c). These findings suggest that the anti-HSC70 antibody strongly prevented the virus from infecting the host cells.

### The HSC70 inhibitor VER155008 inhibits IBDV infection in DF-1 cells

**Fig. 4:**
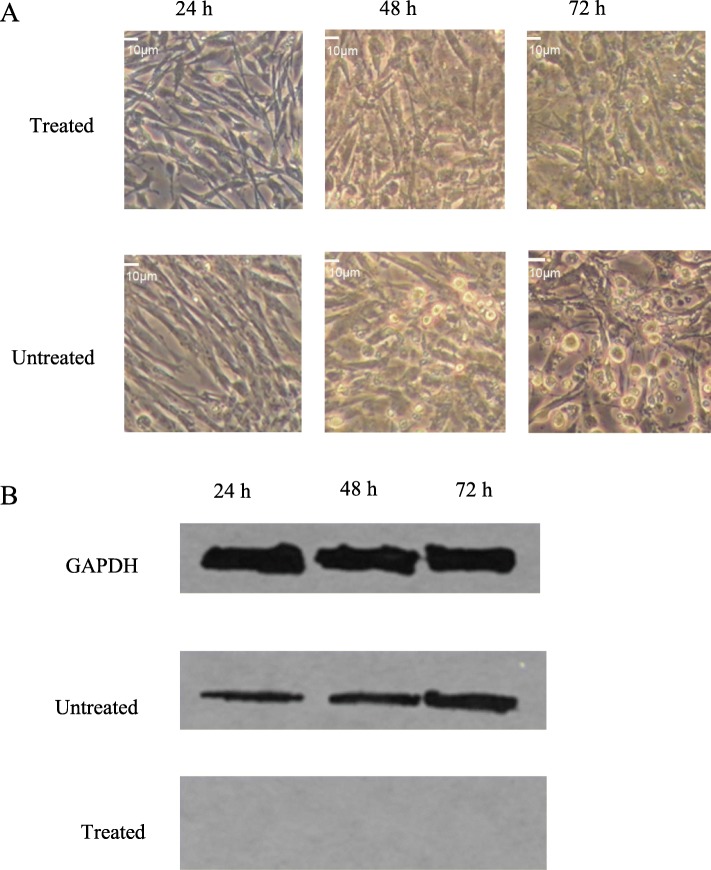
Inhibitory assay of the inhibitor VER155008 for IBDV replication in DF-1 cells. **a**: Morphological changes in DF-1 cells treated with/without the inhibitor VER155008 at different time points. **b**: Western blot results of the VP2 protein in DF-1 cells treated with the inhibitor VER155008 at different time points. GAPDH: internal reference control in each time point

VER155008 is an inhibitor that specifically interacts with HSC70. When we pretreated DF-1 cells with VER155008, no CPE was found in DF-1 cells infected with IBDV, whereas the control group of DF-1 cells infected with IBDV without VER155008 treatment showed abundant CPE (Fig. 4a). This result indicates that VER-155008 inhibited HSC70 and caused viral infection failure in DF-1 cells. Western blot results (Fig. 4b) showed that the expression level of the VP2 protein in the untreated group increased over time; however, the protein expression in the pretreated group could not be detected at different time points, even at 72 h post infection. A viral titer assay (Table 2) showed that the TCID_50_ of the pretreated group was 0 at 24, 48, and 72 h; the TCID_50_ of the untreated group gradually increased over time. This result indicates that VER-155008, an HSC70 inhibitor, prevents infection by the virus.

**Table 2:** The viral titers in DF-1 cells treated with the inhibitor (VER155008)

Viral titer (log_10_TCID_50_/mL)			
Time	24 h	48 h	72 h
Treated	0	0	0
Untreated (control)	3.15	5.94	7.23

### The inhibitory effect of the inhibitor VER-155008 is dose dependent

**Fig. 5:**
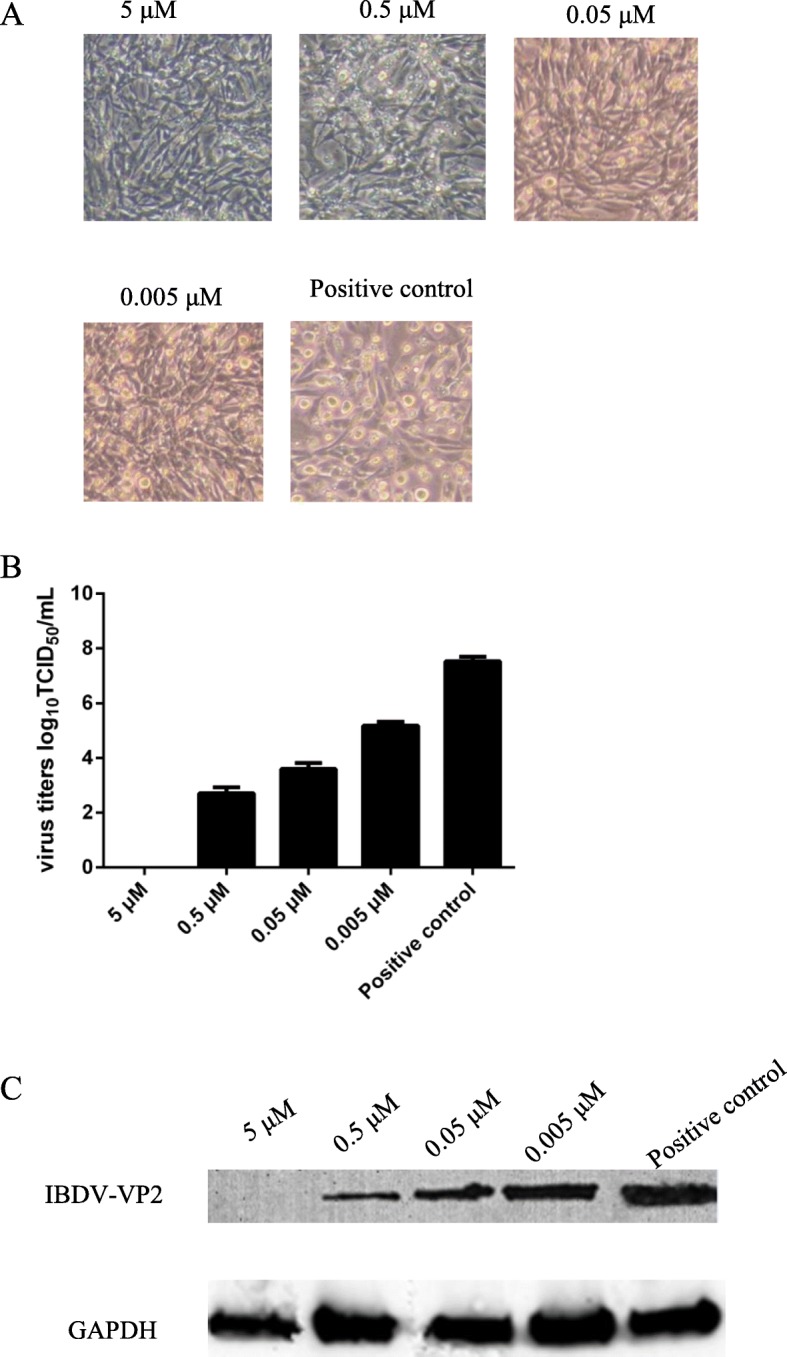
The inhibitory effects on IBDV in DF-1 cells treated with the inhibitor (VER155008) at different concentrations. **a**: The CPE of IBDV in DF-1 cells preincubated with the inhibitor (VER155008) at different concentrations. Positive control: DF-1 cells infected with 0.1 MOI of IBDV. **b**: TCID_50_ titration of IBDV in DF-1 cells pretreated with different concentrations of the inhibitor. **c**: Western blot analysis of IBDV VP2 protein expression in DF-1 cells pretreated with different concentrations of the inhibitor. Lanes 1–4: 5 μM, 0.5 μM, 0.05 μM, and 0.005 μM of VER155008 pretreatment respectively; lane 5: Positive control (DF-1 cells infected with 0.1 MOI of IBDV)

DF-1 cells treated with different concentrations of the inhibitor VER155008 displayed different CPE levels. When the concentration of the inhibitor increased from 0.005 μM to 5 μM, the CPE became weaker to the point where there was almost no effect (Fig. 5). Moreover, the inhibitory effect on IBDV infection increased significantly with the increase in inhibitor concentration. The same results were found for the viral titer and expression level of the IBDV VP2 protein, as shown in Fig. 5a. Figure 5b illustrates that the viral titer gradually decreased with increasing inhibitor concentration compared with that in the positive control group. Surprisingly, as depicted in Fig. 5c, that the expression level of the IBDV VP2 protein was consistent with the variation in viral titer.

## Discussion

In this work, the VP2 protein expressed in the pcDNA-VP2-DF-1 cell line was coprecipitated with the monoclonal antibody 2H11 against the IBDV VP2 protein. At the same time, the proteins interacting with the IBDV VP2 capsid protein were pulled down. Immunoprecipitation and mass spectrometry analysis showed that many proteins on DF-1 cells are able to interact with VP2, including cytoskeleton-related proteins, enzyme-related proteins, and cell regulatory-related proteins. Most of these proteins have been reported to play a receptor role for certain viruses [23, 24]. However, it remains to be further explored whether a protein or a number of proteins synergistically act as receptors. Some reports have revealed that HSC70 may have an important function in virus infection [25, 26].

Interaction between HSC70 and IBDV was corroborated by multiple methods. First, a specific antibody against HSC70 was used to block HSC70 on DF-1 cells. The inhibitory effect on IBDV infection was then investigated by TCID_50_ determination, confocal microscopy and western blotting. The results showed that the anti-HSC70 antibody significantly inhibits viral replication and reduce the viral titer. An assay with an HSC70 inhibitor (VER155008) confirmed that the inhibitor disrupts HSC70 and causes DF-1 cells to lose IBDV adsorption capacity. Failure of the infection showed that the HSC70 inhibitor (VER155008) has a good IBDV blocking effect.

In this study, IBDV particle formation was strongly reduced when HSC70 was blocked by the antibody against HSC70. Preincubation with antibodies resulted in a loss of virus adsorptive capacity, which seriously affected the viral life cycle and led to a sharp decline in viral infection and replication. Protein expression, indirect IFA, and western blot analyses showed that the anti-HSC70 antibody blocked in advance the interaction between the virus and HSC70 on target cells, that the ability of the virus to bind to target cells was reduced, and that the ability of the virus to infect target cells was also decreased.

VER-155008, an adenosine derivative compound, has the characteristics of an ATP analog, with similar strong affinity and activity toward the HSP70 family as ATP. Thus, VER-155008 can be used as a competitive inhibitor [27] . Some researchers have found that the isoform of HSP70 in Kaposi’s sarcoma-associated herpesvirus infection plays a critical role in replication; viral infectivity of host cells declined sharply when the inhibitor VER-155008 was applied [26]. Autographacalifornica nuclear polyhedrosis virus has very strong infectivity toward the grass armyworm, and Lyupina et al. showed that infectivity of this virus is associated with HSC70. Additionally, viral protein synthesis, replication, genome and particle release were severely inhibited when VER-155008 was added in the early stages [28]. Some scholars have found that HSP70 plays an important role in dengue virus infection and replication, whereby the invasive ability of the virus decreased when target cells were preincubated with inhibitors [29]. VER-155008, a specific inhibitor, is generally used for HSC70 competitive inhibition tests and prevents hydrolysis catalyzed by the ATPase domain of HSC70, resulting in conformational changes in the HSC70 protein that make it impossible for IBDV to recognize HSC70 properly and interfering with the specific binding of IBDV to the HSC70 receptor protein; this hinders the infection and replication of the virus. Moreover, the VER155008 inhibited viral infection in DF-1 cells in a dose-dependent manner. When the drug concentration was increased 10-fold from 0.005 μM to 5 μM, the inhibitory effect gradually strengthened. At 5 μM, viral replication and infection could not be detected based on CPE, viral titer and VP2 protein expression. Overall, a marked inhibitory effect was observed.

Beside of HSC70, other proteins in Table 1 may play some role in infection of the virus. It has been reported that human cytomegalovirus mediates and enhances infection of host cells by stimulating the activity of serine/threonine protein phosphatase [30]. Simian vacuolar virus (SV 40) can infect host cells by activating phosphoinositide phospholipase C. If phosphoinositide phospholipase C is blocked, virus infection is inhibited [23]. Chikungunya virus is a mosquito-transmitted virus that binds to ATP synthase subunit beta on insect cells and then enters the cells, resulting in successful infection [24]. When vimentin on the cell surface is reduced by artificial interference, the invasiveness of enterovirus 71 in cells is significantly decreased. Therefore, it is believed that vimentin is an important adsorption factor [31]. Nonetheless, the role of these proteins in viral infection needs to be further studied.

## Conclusions

To the best of our knowledge, this study demonstrates for the first time that HSC70 can interact with the IBDV VP2 protein and promote infection. Such knowledge of the interaction between HSC70 and IBDV will allow for a better understanding of the pathogenesis of IBDV.

## Data Availability

All data generated or analyzed during this study are included in this published article.

## Abbreviations

ACN: acetonitrile
BSA: bovine serum albumin
CPE: cytopathic effect
DMEM: Dulbecco’s modified Eagle’s medium
DTT: DL-Dithiothreitol
FA: formic acid
FBS: fetal bovine serum
FITC: fluoresceine isothiocyanate
GRP78: glucose-regulated protein 78
HSC70: heat shock cognate 70
HSP70: heat shock protein 70
HSV: herpes simplex virus
IAA: iodoacetamide
IBDV: infectious bursal disease virus
IFA: immunofluorescence assay
MOI: multiplicity of infection
PBS: phosphate-buffered saline
NP-40: Nonidet P40
SDS: sodium dodecyl sulfate
SDS-PAGE: sodium dodecyl sulfate polyacrylamide gel electrophoresis
TCID50: 50% tissue culture infective dose
TFA: trifluoroacetic acid
VOPBA: virus overlay protein-binding assay
